# Effectiveness of Managing Cancer and Living Meaningfully Therapy on Health‐Related Outcomes for Patients With Cancer: A Systematic Review and Meta‐Analysis of Randomized Controlled Trials

**DOI:** 10.1111/wvn.70023

**Published:** 2025-04-28

**Authors:** Yizhen Zhang, Xu Zhang, Xiaoming Zhang, Yifan Duan, Yanling Tao

**Affiliations:** ^1^ Shenzhen Clinical Medical College Guangzhou University of Chinese Medicine Shenzhen China; ^2^ Department of Nursing Longgang Central Hospital of Shenzhen Shenzhen China; ^3^ Department of Spine Surgery Chenzhou 3RD People's Hospital Chenzhou China; ^4^ Department of Emergency The People's Hospital of Baoan Shenzhen Shenzhen China; ^5^ Department of Orthopedic Sports Medicine Shenzhen Pingle Orthopedic Hospital, Shenzhen Pingshan Traditional Chinese Medicine Hospital Shenzhen China

**Keywords:** cancer, managing cancer and living meaningfully, meta‐analysis, psychological distress, quality of life

## Abstract

**Background:**

Managing cancer and living meaningfully (CALM) therapy is a psychosocial intervention designed to enhance the well‐being of cancer patients; however, its impact on health‐related outcomes in cancer patients remains unclear.

**Aims:**

This study aimed to systematically synthesize current evidence to assess the impact of CALM therapy on health‐related health outcomes and identify key features for optimizing the intervention.

**Methods:**

An exhaustive search was conducted across seven databases from inception to July 5, 2024. Two reviewers separately evaluated the eligibility of studies, performed data extraction, and examined the methodological quality using the Cochrane's risk of bias tool. The certainty of evidence was evaluated using GRADE, and data analysis was conducted with Review Manager 5.4. The study protocol was registered with PROSPERO (CRD42024568561).

**Results:**

Eleven studies (*n* = 1284) were included. CALM therapy showed significant improvements in psychological distress (MD = −2.43, 95% CI [−3.99, −0.86], *p* = 0.002), anxiety (SMD = −1.06, 95% CI [−1.78, −0.34], *p* = 0.004), depression (SMD = −0.65, 95% CI [−1.13, −0.17], *p* = 0.008), quality of life (SMD = 1.44, 95% CI [0.47, 2.40], *p* = 0.003), cancer‐related fatigue (SMD = −3.54, 95% CI [−5.84, −1.23], *p* = 0.003), and sleep disturbance (SMD = −1.00, 95% CI [−1.86, −0.14], *p* = 0.02). However, its effects on alleviating the fear of cancer recurrence were not statistically significant.

**Linking Evidence to Action:**

CALM therapy has demonstrated positive effects on psychological distress, anxiety, depression, cancer‐related fatigue, sleep disturbance, and quality of life. However, evidence regarding its effects on the fear of cancer recurrence remains limited. While we explored potential sources of heterogeneity, no primary cause was identified. Our findings remained largely consistent after this exploration, but due to the observed heterogeneity, these results should still be interpreted with caution. Further high‐quality randomized controlled trials with larger sample sizes are needed to confirm these findings and assess the long‐term implications of CALM therapy.

## 1 Introduction

The most recent estimates from the International Agency for Research on Cancer (IARC) indicated that nearly 20 million new cancer diagnoses and 10 million cancer‐related deaths were reported in 2022. Global cancer incidence is projected to rise by 77% by 2050, reaching 35 million, thereby presenting significant challenges to healthcare systems worldwide (Bray et al. 2024). In addition to a range of debilitating physical symptoms, such as pain, fatigue, and sleep issues, a cancer diagnosis and its treatment significantly affects various aspects of patients' lives. These effects often result in increased psychological difficulties, driven by psychological, social, and spiritual factors, and commonly manifest as fear of cancer recurrence, anxiety, depression, and emotional distress (Cillessen et al. 2019; Wang et al. 2023). Studies indicate that approximately 20%–54% of cancer patients suffer from clinically significant psychological problems, which include distress (Mehnert et al. 2018), severe anxiety, depression symptoms, and feelings of demoralization or uncertainty (Kim et al. 2011; Rao et al. 2019; Subramaniam et al. 2018). However, psychological disturbances and cancer‐related complications often interact with one another. Research has shown that these psychological challenges not only reduce cancer patients' adherence to treatment but also negatively impact their quality of life and increase mortality risk. Consequently, this can result in increased healthcare utilization, higher medical costs, and significant economic losses (Karunanithi et al. 2018; McDermott et al. 2018; Smith 2015; Van Beek et al. 2021). The co‐occurrence of both physical and mental symptoms in cancer patients presents significant challenges to patients and their healthcare providers. As a result, interventions aimed at improving health‐related outcomes for this group are of critical importance.

Advances in healthcare have extended the survival of cancer patients, and early implementation of palliative care has been demonstrated to improve psychological health, quality of life, and survival outcomes (Sethi et al. 2020). The managing cancer and living meaningfully (CALM) intervention developed by Rodin et al. (2018), is a well‐structured, short‐term psychosocial approach designed to address psychological disorders and improve cancer patients' overall well‐being. The therapy targets four key aspects: (1) managing symptoms while fostering effective communication with healthcare professionals, (2) re‐evaluating personal identity and interpersonal relationships, (3) fostering resilience in facing life's uncertainties and the future, and (4) enhancing spiritual well‐being through finding meaning (Caruso et al. 2020). Over the past few years, a growing body of clinical studies has examined the impact of CALM therapy on various health outcomes in cancer patients. Numerous studies have demonstrated that this intervention effectively reduces psychological distress, anxiety, and depressive symptoms; alleviates fear of recurrence; and enhances the quality of life among cancer patients. A recent systematic review synthesized relevant studies in the field, confirming the effectiveness of CALM therapy on advanced cancer patients (Wulandari et al. 2020). However, the inclusion of non‐randomized controlled trials (RCTs) and pilot studies reduced the scientific rigor of the review, and no data synthesis was conducted.

Therefore, we performed this systematic review and meta‐analysis of RCTs to evaluate the effectiveness of CALM therapy on psychological (psychological distress, anxiety, and depression) and physical (cancer‐related fatigue and sleep disturbances) outcomes, along with health‐related quality of life, by comprehensively reviewing existing studies on its application in cancer patients. Additionally, our review aimed to identify the key characteristics of effective CALM therapy to facilitate evidence dissemination and support the implementation of personalized care strategies in clinical practice.

## 2 Methods

This systematic review was performed in compliance with the PRISMA‐2020 guidelines (Page et al. 2021) and was registered in PROSPERO on August 13, 2024 (CRD42024568561).

### 2.1 Search Strategy

A systematic search was performed in PubMed, EMBASE, Web of Sciences, Cochrane Central Register of Controlled Trials, CINAHL (EBSCOhost), and PsycINFO (EBSCOhost) from inception to July 5, 2024. Google Scholar, Clinical Trials.gov, the reference lists of included studies, and existing systematic reviews on CALM therapy were also retrieved. The search strategy and eligibility criteria were formulated based on the Population, Intervention, Comparison, Outcomes, and Study Design (PICOS) framework. The PICOS question that guided the review is detailed in Table 1, along with the eligibility criteria. Additionally, the details of the search strategy can be found in Table S1.

**TABLE 1:** Eligibility criteria in this study.

PICOS components	Description
**Inclusion criteria**	
P (population)	Adult patients or survivors (≥ 18 years) with a diagnosis of cancer with any type and in any stage
I (intervention)	Managing Cancer and Living Meaningfully (CALM) intervention
C (Comparison)	Conventional therapy or routine care
O (outcomes)	Primary outcomes include psychological distress, anxiety, depression and quality of life; Secondary outcomes including fear of cancer recurrence, cancer‐related fatigue and sleep quality
S (study design)	Randomized controlled trials
**Exclusion criteria**	
Study protocols, letters, comments, editorials, and conference abstracts, studies with incomplete or unusable data and cannot be obtained after contacting authors.	

### 2.2 Study Selection and Data Extraction

Duplicates in the retrieved articles were identified and removed using NoteExpress software. Two reviewers then independently screened the studies based on the predefined eligibility criteria. The third reviewer was consulted to solve disagreements until there was a consensus. The data extraction table included (1) general information about studies, including the first author, country, and publication year; (2) participant characteristics, including the type of patients, sample size, and sample attrition, age, and sex; (3) intervention characteristics, including intervention duration and implementer; (4) control characteristics; and (5) measures and outcomes and data collection time‐points.

### 2.3 Quality Assessment

#### 2.3.1 Methodological Quality

The Cochrane risk bias assessment tool including seven aspects (Higgins et al. 2011) was utilized to evaluate the methodological quality. Two reviewers conducted independent assessments of the risk of bias, with any disagreements addressed through discussion or, if necessary, by consulting a third reviewer to reach a consensus.

#### 2.3.2 Certainty of Evidence

Certainty in the pooled evidence was assessed using the Grading of Recommendations Assessment, Development, and Evaluation (GRADE) method within GRADEpro GDT (https://gradepro.org), addressing five domains: (1) risk of bias, (2) inconsistency, (3) imprecision, (4) indirectness, and (5) publication bias (Ryan and Hill 2016).

### 2.4 Data Analysis

Statistical analysis was conducted using Review Manager 5.4. A meta‐analysis assessed the pooled effects of CALM on patient‐reported outcomes between intervention and control groups at post‐intervention and follow‐up, contingent on data from at least three studies. Continuous variables were represented as mean differences (MDs) or standardized mean differences (SMDs) with 95% confidence intervals (CIs), depending on the measurement scale used. SMDs of 0.2, 0.5, and 0.8 indicate small, moderate, and large effects, respectively (Andrade 2020). Heterogeneity was evaluated with the χ^2^ test (*p <* 0.1 considered significant) and the *I* ^2^ statistic. A fixed‐effects model was applied for low heterogeneity (*p* > 0.1, *I* ^2^ < 50%) (Higgins et al. 2003), while a random‐effects model was used otherwise, alongside subgroup analyses to identify sources of heterogeneity. Statistical significance was defined as *p* < 0.05. Heterogeneity levels were categorized as 0%, 25%, 50%, and 75% for no, low, moderate, and high heterogeneity, respectively. Sensitivity analysis utilized the leave‐one‐out approach, and publication bias was evaluated by examining funnel plots visually.

## 3 Results

### 3.1 Study Selection

**FIGURE 1:**
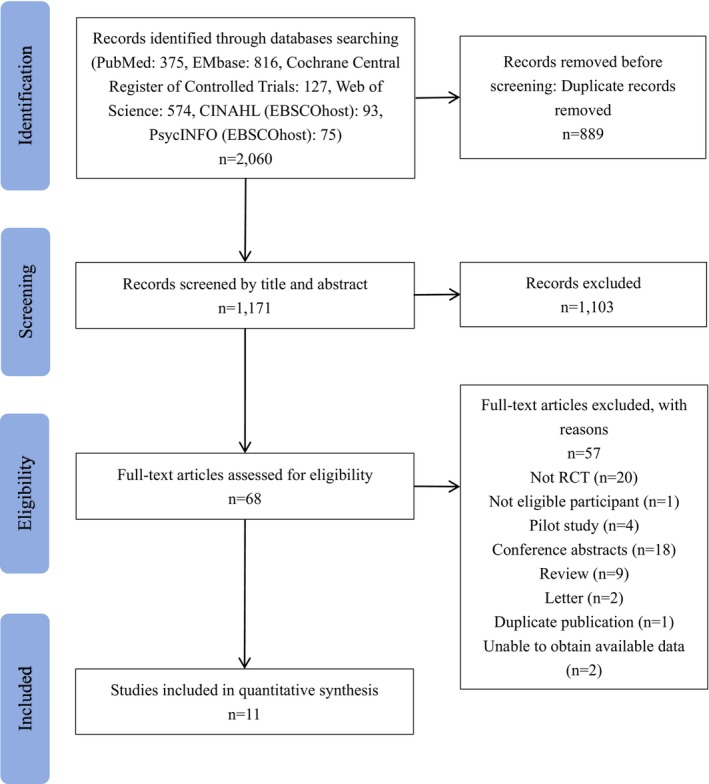
Flowchart of study selection and literature screening process.

A total of 2060 records were retrieved, and after removing duplicate studies, 1103 studies were excluded by screening titles and abstracts. Following the initial screening, 68 studies were evaluated for eligibility, with 54 excluded for various reasons: non‐RCTs (*n* = 20), ineligible participants (*n* = 1), pilot studies (*n* = 4), conference abstracts (*n* = 18), reviews (*n* = 9), letters (*n* = 2), duplicates (*n* = 1), or lack of data (*n* = 2). Finally, 11 RCTs (including 1284 patients) satisfied the eligibility criteria and were presented in Figure 1.

### 3.2 Study Characteristics and Results

This review encompassed 11 RCTs published from 2018 to 2023, with nine conducted in China (Cai, Zhang, et al. 2023; Cai, Zhao, et al. 2023; Ding et al. 2020; Jing et al. 2022; Liu et al. 2023; Pang et al. 2023; Wang et al. 2023; Zhang, Yao, et al. 2022; Zhao et al. 2023), and one each conducted in Germany (Mehnert et al. 2020) and Canada (Rodin et al. 2018). A total of 1284 cancer patients were enrolled in the included studies, with 632 in the experimental groups and 652 in the control groups. Sample sizes varied between 60 and 227. Two studies recruited patients with mixed cancer types (Mehnert et al. 2020; Rodin et al. 2018), and nine studies recruited patients with a single type of cancer, including esophageal (Cai, Zhang, et al. 2023), breast (Ding et al. 2020; Liu et al. 2023; Pang et al. 2023; Wang et al. 2023; Zhang, Yao, et al. 2022), gastrointestinal (Cai, Zhang, et al. 2023; Jing et al. 2022), and lung (Zhao et al. 2023) cancers. The participants' mean age varied from 49.26 years (SD = 10.116) to 68.56 years (SD = 9.55), with an overall weighted mean age of 58.29 years. Males comprised 58.4% of the total sample. All of the included studies covered the four core areas of CALM therapy. The number of intervention sessions ranged from three to six sessions, spanning 3–8 months, with each session lasting between 30 and 70 min. Additional study characteristics are detailed in Table S2.

### 3.3 Quality Assessment and Risk of Bias

As depicted in Figure S1, eight studies (Cai, Zhang, et al. 2023; Ding et al. 2020; Jing et al. 2022; Liu et al. 2023; Mehnert et al. 2020; Rodin et al. 2018; Zhang, Yao, et al. 2022; Zhao et al. 2023) were assessed as having a low risk of bias in random sequence generation and allocation concealment methods, and three studies (Cai, Zhao, et al. 2023; Pang et al. 2023; Wang et al. 2023) did not provide clear details on the random allocation method and were evaluated as having an unclear risk of bias. Additionally, eight studies (Cai, Zhang, et al. 2023; Ding et al. 2020; Jing et al. 2022; Liu et al. 2023; Mehnert et al. 2020; Rodin et al. 2018; Zhang, Yao, et al. 2022; Zhao et al. 2023) were identified as having a high risk of bias due to the lack of blinding of participants and interventionists. Only one study (Cai, Zhang, et al. 2023) that described the allocation concealment methods of outcome assessment was considered as having a low risk of bias. Additional details regarding the evaluation are presented in Table S3. The evidence certainty for the outcomes was evaluated as low, with a summary presented in Table S4 following the GRADE criteria.

### 3.4 Synthesis of Results

#### 3.4.1 Primary Outcomes

##### 3.4.1.1 Psychological Distress

**FIGURE 2:**
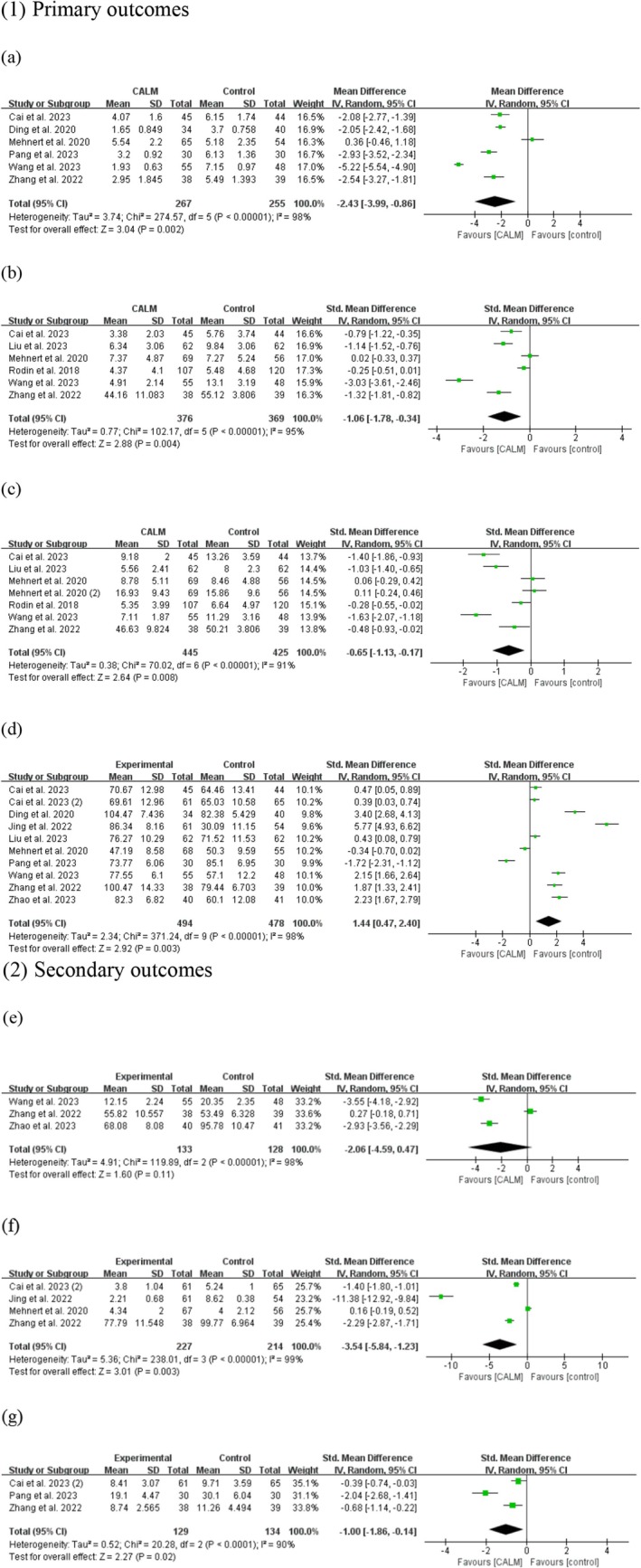
Effects of CALM therapy on primary and secondary outcomes: (a) Psychological distress; (b) Anxiety; (c) Depression; (d) Quality of life; (e) Fear of cancer recurrence; (f) Cancer‐related fatigue; (g) Sleep disturbance.

Six studies (Cai, Zhao, et al. 2023; Ding et al. 2020; Mehnert et al. 2020; Pang et al. 2023; Wang et al. 2023; Zhang, Yao, et al. 2022) reported on the impact of CALM therapy on psychological distress in cancer patients, involving a total of 522 participants. The pooled mean difference (MD) revealed a significant effect of CALM therapy on decreasing psychological distress in comparison with the control group (MD = −2.43, 95% CI [−3.99, −0.86], *p* = 0.002) (Figure 2a). Significant heterogeneity was observed among these studies (*I* ^2^ = 98%, *p* < 0.001).

##### 3.4.1.2 Anxiety

Six studies involving 745 patients (Cai, Zhao, et al. 2023; Liu et al. 2023; Mehnert et al. 2020; Rodin et al. 2018; Wang et al. 2023; Zhang, Yao, et al. 2022) measured the effects of CALM therapy on anxiety in cancer patients. The pooled findings revealed that the anxiety level of the intervention group was significantly lower than that of the control group (SMD = −1.06, 95% CI [−1.78, −0.34], *p* = 0.004) (Figure 2b), but high heterogeneity was detected (*I* ^2^ = 95%, *p* < 0.001).

##### 3.4.1.3 Depression

Six studies (Cai, Zhao, et al. 2023; Liu et al. 2023; Mehnert et al. 2020; Rodin et al. 2018; Wang et al. 2023; Zhang, Yao, et al. 2022) measured the CALM intervention effects on depression, with a total of 745 patients, from which we extracted seven sets of data for analysis. The pooled results indicated a significant decrease in depression symptoms following CALM intervention (SMD = −0.65, 95% CI [−1.13, −0.17], *p* = 0.008) (Figure 2c). A high level of heterogeneity was observed among these studies (*I* ^2^ = 91%, *p* < 0.001).

##### 3.4.1.4 Quality of Life

The effectiveness of CALM therapy on quality of life in cancer patients was reported by 10 studies (Cai, Zhang, et al. 2023; Cai, Zhao, et al. 2023; Ding et al. 2020; Jing et al. 2022; Liu et al. 2023; Mehnert et al. 2020; Pang et al. 2023; Wang et al. 2023; Zhang, Yao, et al. 2022; Zhao et al. 2023), with a total of 972 patients. The pooled findings indicated that CALM therapy significantly enhanced the quality of life in cancer patients compared to the control group (SMD = 1.44, 95% CI [0.47, 2.40], *p* = 0.003) (Figure 2d). However, a high level of heterogeneity was noted among these studies (*I* ^2^ = 98%, *p* < 0.001).

#### 3.4.2 Secondary Outcomes

##### 3.4.2.1 Fear of Cancer Recurrence

Three studies (Wang et al. 2023; Zhang, Yao, et al. 2022; Zhao et al. 2023) involving 261 cancer patients measured fear of cancer recurrence. The meta‐analysis indicated no significant difference in reducing fear of recurrence between the CALM therapy group and the control group (SMD = −2.06, 95% CI [−4.59, 0.47], *p* = 0.11) (Figure 2e). High heterogeneity was observed in these studies (*I* ^2^ = 98%, *p* < 0.001).

##### 3.4.2.2 Cancer‐Related Fatigue

Four studies (Cai, Zhang, et al. 2023; Jing et al. 2022; Mehnert et al. 2020; Zhang, Yao, et al. 2022) involving 441 cancer patients reported cancer‐related fatigue. The meta‐analysis results indicated that CALM therapy significantly reduced cancer‐related fatigue compared to usual care (SMD = −3.54, 95% CI [−5.84, −1.23], *p* = 0.003) (Figure 2f). However, there remains a high level of heterogeneity among these studies (*I* ^2^ = 99%, *p* < 0.001).

##### 3.4.2.3 Sleep Disturbance

Three studies (Cai, Zhang, et al. 2023; Pang et al. 2023; Zhang, Yao, et al. 2022) involving 263 cancer patients reported on sleep quality. The pooled results indicated that CALM therapy could improve cancer patients' sleep disturbance (SMD = −1.00, 95% CI [−1.86, −0.14], *p* = 0.02) (Figure 2g). High heterogeneity was observed across these studies (*I* ^2^ = 90%, *p* < 0.001).

### 3.5 Subgroup Analyses

Subgroup analysis showed that when the CALM intervention lasted less than 3 months, there was no significant effect on the quality of life in cancer patients (SMD = −0.08, 95% CI [−0.87, 0.72], *p* = 0.85). Both sessions duration shorter or longer than 30 min were effective in reducing distress, anxiety, and depression. At the 1‐month follow‐up after the CALM intervention ended, there was a non‐significant improvement in the quality of life (SMD = 1.39, 95% CI [−0.04, 2.83], *p* = 0.06). Additional detailed findings from the subgroup analyses are provided in Table 2.

**TABLE 2:** Subgroup analysis of the pooled effects on primary outcomes.

Primary outcomes	Number of studies	Number of participants (I/C)	MD/SMD (95% CI)	*I* ^*2*^(%)	*p*
**Intervention duration**					
(1) *Distress*					
≤ 3 months	3	113/113	−2.54 (−3.04, −2.04)^a^	40	< 0.001
≥ 6 months	3	154/142	−2.32 (−5.18, 0.53)^a^	99	0.11
(2) *Anxiety*					
≤ 3 months	2	107/106	−0.98 (−1.32, −0.64)	30	< 0.001
≥ 6 months	4	269/263	−1.12 (−2.23, −0.01)	97	0.05
(3) *Depression*					
≤ 3 months	2	107/106	−1.19 (−1.54, −0.83)	31	< 0.001
≥ 6 months	5	338/319	−0.43 (−0.96, 0.10)	91	0.12
(4) *Quality of life*					
≤ 3 months	7	198/201	−0.08 (−0.87, 0.72)	93	0.85
≥ 6 months	3	296/277	2.49 (0.95, 4.03)	98	0.002
**Duration of each session**					
(1) *Distress*					
≤ 30 min	2	72/79	−2.19 (−2.62, −1.76)^a^	27	< 0.001
≥ 30 min	4	195/176	−2.49 (−4.82, −0.15)^a^	99	0.04
(2) *Anxiety*					
≤ 30 min	1	62/62	−1.14 (−1.52, −0.76)	/	< 0.001
≥ 30 min	5	314/307	−1.05 (−1.92, −0.18)	96	0.02
(3) *Depression*					
≤ 30 min	1	62/62	−1.03 (1.40, −0.65)	/	< 0.001
≥ 30 min	6	383/363	−0.59 (−1.12, −0.05)	92	0.03
(4) *Quality of life*					
≤ 30 min	4	197/197	2.94 (0.80, 5.07)	98	0.007
≥ 30 min	6	297/281	0.47 (−0.49, 1.43)	96	0.34
**Scoring criteria**					
(1) *Anxiety*					
HAD‐A	3	162/154	−1.64 (−2.82, −0.45)	95	0.007
GAD‐7	2	176/176	−0.14 (−0.40, 0.12)	31	0.28
SAS	1	38/39	−1.32 (−1.81, −0.82)	/	< 0.001
(2) *Depression*					
HAD‐D	3	162/154	−1.33 (−1.69, −0.97)	52	< 0.001
PHQ‐9	2	176/176	−0.13 (−0.47, 0.21)	58	0.45
BDI‐II	1	69/56	0.11 (−0.24, 0.46)	/	0.53
SDS	1	38/39	−0.48 (−0.93, v‐0.02)	/	0.04
(3) *Quality of life*					
EORTC‐QLQ‐C30	4	207/204	2.18 (0.38, 3.97)	98	0.02
FACT‐B	4	189/189	1.94 (0.73, 3.15)	96	0.002
QUAL‐EC	1	68/55	−0.34 (−0.70, 0.02)	/	0.06
QOL	1	30/30	−1.72 (−2.31, −1.12)	/	< 0.001
**Follow‐up time**					
(1) *Distress*					
Immediate post‐intervention	4	188/171	−2.60 (−4.89, −0.31)^a^	98	0.03
1‐month follow‐up	2	79/84	−2.06 (−2.38, −1.73)^a^	0	< 0.001
(2) *Anxiety*					
Immediate post‐intervention	4	269/263	−1.12 (−2.23, −0.01)	97	0.05
1‐month follow‐up	2	107/106	−0.98 (−1.32, −0.64)	30	< 0.001
(3) *Depression*					
Immediate post‐intervention	5	338/319	−0.43 (−0.96, 0.10)	91	0.12
1‐month follow‐up	2	107/106	−1.19 (−1.54, −0.83)	31	< 0.001
(4) *Quality of life*					
Immediate post‐intervention	7	353/332	1.46 (0.12, 2.80)	98	0.03
1‐month follow‐up	3	494/478	1.39 (−0.04, 2.83)	98	0.06

Abbreviations: BDI‐II, Beck Depression Inventory; EORTC‐QLQ‐C30, European Organization for Research and Treatment of Cancer Quality of Life Questionnaire Core 30; FACT‐B, functional assessment of cancer therapy‐breast; GAD‐7, generalized anxiety disorder questionnaire; HAD‐A, Anxiety subscale of the Hospital Anxiety and Depression Scale; HAD‐D, Depression subscale of the Hospital Anxiety and Depression Scale; PHQ‐9, patient health questionnaire; I/C, intervention group/control group; QOL; QUAL‐EC, quality of life at the end of life cancer scale; SAS, self‐rating anxiety scale.

^a^ Represents the MD (mean difference).

### 3.6 Sensitivity Analysis and Publication Bias

The results of the sensitivity analysis indicated that the pooled effects of CALM therapy on psychological distress, anxiety, depression, and quality of life remained significant when employing the leave‐one‐out approach, demonstrating the robustness of the pooled estimates. Visual inspection of the funnel plots revealed no evidence of publication bias (Figure S2).

## 4 Discussion

As far as we know, this study represents the first meta‐analysis to measure the direction and strength of the relationships between CALM therapy and health‐related outcomes in cancer patients. Eleven RCTs were included, with pooled results indicating that CALM therapy had significant effects on psychological distress, anxiety, depression, quality of life, cancer‐related fatigue, and sleep disturbance, while showing no significant effect on fear of cancer recurrence.

This review identified considerable heterogeneity, which may stem from variations in cancer types among participants, differences in the duration of interventions, and the diversity of assessment tools used. Moreover, the evidence certainty of included studies was low, largely due to factors such as missing outcome data, significant inter‐study heterogeneity, and limited sample sizes. These factors could compromise the reliability of the results. Consequently, caution is warranted in interpreting the findings of this study.

Studies have found that approximately 52% of cancer patients endure severe psychological distress (Mehnert et al. 2018). This study shows that CALM therapy effectively alleviates psychological distress in cancer patients. Previous studies have established that psychological interventions can effectively reduce psychological distress in cancer patients, particularly mindfulness‐based interventions (Cillessen et al. 2019), meaning therapy (Sun et al. 2024), and acceptance and commitment therapy (Li et al. 2021). Our findings are consistent with this body of evidence, further validating the efficacy of CALM therapy. Furthermore, our subgroup analysis based on follow‐up duration indicated that the efficacy of CALM therapy remained significant 1‐month post‐treatment. However, the conclusions drawn from this study are derived from a limited number of trials, accompanied by considerable heterogeneity among them; thus, caution is warranted in the interpretation of these findings.

Despite advancements in early cancer detection that have extended survival, a cancer diagnosis still imposes a significant psychological burden, with anxiety and depression symptoms respectively affecting up to 30.2% and 22.6% of cancer patients (Shalata et al. 2024). Our study demonstrated that CALM therapy significantly alleviated anxiety and depression symptoms, a finding supported by a systematic review that assessed the effectiveness of psychological treatments in cancer patients (Zhang, Li, and Hu 2022). This therapy may improve patients' psychological well‐being through several mechanisms. First, it encourages emotional expression and communication, allowing patients to voice their concerns and feelings about their illness, which helps reduce internal stress. Second, this method highlights the importance of meaning and purpose in life, aiding patients in reframing their outlook and alleviating symptoms of anxiety and depression (Ding et al. 2020; Sethi et al. 2020). However, the studies examining these two outcomes exhibit considerable heterogeneity, potentially stemming from variations in participant characteristics and the measurement tools used. Consequently, it is essential to interpret these results with caution.

Our study suggested that CALM therapy has no significant impact on alleviating the fear of cancer recurrence in cancer patients. This result differs from a previous meta‐analysis that synthesized evidence from 11 studies, which found that psychological interventions significantly reduced fear of cancer recurrence in breast cancer patients, although the corresponding effect size was relatively small (SMD = −0.31) (Lyu et al. 2022). Among the three studies included in our study, Wang et al. (2023) and Zhao et al. (2023) demonstrated that CALM therapy resulted in a reduction in fear of cancer recurrence among patients. In contrast, Zhang, Yao, et al. (2022) found that while VR‐CALM intervention showed statistically significant changes in fear of cancer recurrence before and after treatment, these differences were not statistically significant when compared to the control group. Outcomes among the studies may differ due to variations in study design, patient populations, intervention delivery methods, and assessment tools used. Due to the limited number of studies examining this outcome and the significant heterogeneity among the three, definitive conclusions cannot be drawn.

Cancer‐related fatigue and sleep disturbance are frequently reported as common and significant symptoms experienced by patients undergoing cancer treatment, often persisting even after the completion of therapy (Wu et al. 2022). Evidence suggests that 77.5% of cancer patients report clinically significant fatigue, and 78% report poor sleep quality. These issues are closely associated with reduced quality of life (Al Maqbali et al. 2021). Our findings indicated that CALM therapy has a statistically significant effect on cancer‐related fatigue and sleep disturbances, which supported the conclusions of a prior review (Ma et al. 2023). This review highlighted the effectiveness of telemedicine‐based psychosocial interventions in improving fatigue, sleep disturbances, distress, quality of life, and sexual function in breast cancer patients, but found no significant impact on anxiety, depression, and fear of cancer recurrence. The primary goal of psychological interventions is to modify cognition, emotions, and behavior, or a combination of these factors, and such interventions may alleviate cancer‐related fatigue (Jing et al. 2022). Nevertheless, this review was limited by the inclusion of only four studies addressing fatigue and three studies on sleep disturbances, with considerable heterogeneity observed across them. Therefore, additional large‐scale studies are necessary to validate these findings.

In addition, our findings demonstrated that CALM therapy significantly improves the overall quality of life for cancer patients, supporting the results of other meta‐analyses that examine psychological interventions in this population (Sun et al. 2024; Zhang et al. 2023). CALM is an evidence‐based and standardized psychosocial intervention that aims to address the practical and existential challenges faced by cancer patients. This therapy addresses the practical and existential challenges faced by cancer patients, alleviates their physical and emotional suffering, aids them in seeking meaning and purpose in life, and ultimately enhances their quality of life (Sethi et al. 2020). Notably, the effect of CALM therapy on quality of life at the 1‐month follow‐up was not statistically significant. Furthermore, due to the considerable heterogeneity among the included studies, caution is warranted when interpreting the findings related to quality of life. Thus, additional large‐scale RCTs are necessary to confirm the long‐term effectiveness of CALM on quality of life.

### 4.1 Limitations

Several limitations in our study should be acknowledged. First, methodological limitations in the included studies cannot be overlooked, such as small sample sizes and the absence of blinding for participants, intervention providers, and outcome assessors. Second, studies included in this review exhibited considerable heterogeneity due to differences in participant demographics, types of cancer, sample sizes, and assessment methods. Third, most studies lack long‐term follow‐up research, which prevents us from confirming the long‐term effects of CALM therapy on cancer patients. Fourth, the majority of included studies in this review were performed in China (9 out of 11), highlighting the necessity for additional research to confirm the effects of the CALM intervention in diverse cultural contexts. Lastly, this review did not consider the impact of CALM therapy on various cancer types. Consequently, further randomized controlled trials should focus on specific cancer patient groups to clarify this matter.

### 4.2 Implications for Future Research and Clinical Practice

This study indicated that CALM therapy significantly contributes to improving health‐related outcomes for cancer patients. However, the conclusions derived from the current body of research necessitate further validation. First, the limited number of RCTs included in this study underscores the imperative for additional high‐quality trials with larger sample sizes. Second, future studies should aim to elucidate the intervention effects across diverse populations and incorporate long‐term follow‐up and economic impact analyses. Furthermore, it is essential for upcoming research to establish robust evidence regarding the optimal intervention format, dosage, and duration.

Our findings highlight the promise of integrating CALM therapy as an essential component of comprehensive care for cancer patients. Healthcare professionals should contemplate incorporating CALM therapy into standard treatment protocols to effectively address the physiological and psychological health requirements of this population. Given that CALM therapy generally involves only an initial face‐to‐face session with patients, followed by subsequent treatments conducted via phone or video, it can be readily adopted by a diverse range of trained healthcare professionals (Sethi et al. 2020). Furthermore, since it does not impose strict psychological qualifications on implementers and is highly scalable (Rodin and Hales 2021), training healthcare providers, especially nurses, in its application can significantly enhance their ability to help patients manage both psychological and physical distress while discovering meaning in their lives. Additionally, promoting interdisciplinary collaboration may enhance the delivery of CALM therapy, ensuring that patients receive tailored, holistic care that meets their specific needs.

## 5 Conclusions

In conclusion, this systematic review and meta‐analysis includes 11 RCT and confirms that CALM therapy may have potential effectiveness in improving psychological health (psychological distress, anxiety, and depression), physical symptoms (cancer‐related fatigue and sleep disturbances), and health‐related quality of life of cancer patients. However, the evidence regarding its impact on the fear of cancer recurrence is insufficient. Additionally, the limited number of studies for each outcome and the lack of follow‐up assessments indicate the need for more high‐quality, large‐sample randomized controlled trials to validate our findings and elucidate the long‐term effects of CALM therapy in future research.

## Conflicts of Interest

The authors declare no conflicts of interest.

## Supporting information


Data S1.

## Data Availability

The data of the research are available in the tables of the manuscript or on request from the corresponding author.
