# Supplementary material for: Effectiveness of Managing Cancer and Living Meaningfully Therapy on Health‐Related Outcomes for Patients With Cancer: A Systematic Review and Meta‐Analysis of Randomized Controlled Trials
Source: Worldviews Evid Based Nurs. 2025 Apr 28;22(2):e70023. doi: 10.1111/wvn.70023 (PMC12035791; doi:10.1111/wvn.70023)
Supplement: Supplementary file 1 — Data S1. [file WVN-22-0-s001.docx]

**Table S1**

*Search Strategy by Database.*

| **Database** | **Search Strategy** | **Results** |
| --- | --- | --- |
| PubMed | #1 "neoplasms"[MeSH Terms]  #2 "carcinoma"[MeSH Terms]  #3 "cancer*"[Title/Abstract] OR "tumor*"[Title/Abstract] OR "tumour*"[Title/Abstract] OR "neoplasm*"[Title/Abstract] OR "malignan*"[Title/Abstract] OR "carcinoma*"[Title/Abstract] OR "oncology"[Title/Abstract] OR "adenocarcinoma"[Title/Abstract]  #4 #1 OR #2 OR #3  #5 "managing cancer and living meaningfully"[Title/Abstract] OR "CALM"[Title/Abstract]  #6 #4 AND #5 | 375 |
| EMbase | #1 'neoplasm'/exp  #2 'carcinoma'/exp  #3 'cancer*':ab,ti OR 'tumor*':ab,ti OR 'tumour*':ab,ti OR 'neoplasm*':ab,ti OR 'malignan*':ab,ti OR 'carcinoma*':ab,ti OR 'oncology':ab,ti  #4 #1 OR #2 OR #3  #5 'managing cancer and living meaningfully':ab,ti OR 'CALM':ab,ti  #6 #4 AND #5 | 816 |
| Cochrane Central Register of Controlled Trials | #1 MeSH descriptor: [Neoplasms] explode all trees  #2 MeSH descriptor: [Carcinoma] explode all trees  #3 (cancer*):ti,ab,kw OR (tumor*):ti,ab,kw OR (tumour*):ti,ab,kw OR (neoplasm*):ti,ab,kw OR (malignan*):ti,ab,kw OR (carcinoma*):ti,ab,kw OR (oncology):ti,ab,kw OR (adenocarcinoma):ti,ab,kw  #4 #1 OR #2 OR #3  #5 (managing cancer and living meaningfully):ti,ab,kw OR (CALM):ti,ab,kw  #6 #4 AND #5 | 127 |
| Web of Science | #1 TI= (cancer* OR tumor* OR tumour* OR neoplasm* OR malignan* OR carcinoma* OR oncology OR adenocarcinoma)  #2 AB= (cancer* OR tumor* OR tumour* OR neoplasm* OR malignan* OR carcinoma* OR oncology OR adenocarcinoma)  #3 #1 OR #2  #4 TI= ("managing cancer and living meaningfully" OR CALM)  #5 AB= ("managing cancer and living meaningfully" OR CALM)  #6 #4 OR #5  #7 #3 AND #6 | 574 |
| CINAHL (EBSCOhost) | #1 MH "Neoplasms"  #2 MH "Carcinoma"  #3 SU (cancer or cancers or tumor or tumors or tumour or tumours or neoplasm or neoplasms or malignant or malignancy or malignancies or oncology or carcinoma or adenocarcinoma)  #4 AB (cancer or cancers or tumor or tumors or tumour or tumours or neoplasm or neoplasms or malignant or malignancy or malignancies or oncology or carcinoma or adenocarcinoma)  #5 #1 OR #2 OR #3 OR #4  #6 AB managing cancer and living meaningfully OR AB CALM  #7 #5 AND #6 | 93 |
| PsycINFO (EBSCOhost) | #1 DE "Neoplasms"  #2 SU (cancer or cancers or tumor or tumors or tumour or tumours or neoplasm or neoplasms or malignant or malignancy or malignancies or oncology or carcinoma or adenocarcinoma)  #3 AB (cancer or cancers or tumor or tumors or tumour or tumours or neoplasm or neoplasms or malignant or malignancy or malignancies or oncology or carcinoma or adenocarcinoma)  #4 #1 OR #2 OR #3  #5 SU ( managing cancer and living meaningfully ) OR SU CALM  #6 AB ( managing cancer and living meaningfully ) OR AB CALM  #7 #5 OR #6  #8 #4 AND #7 | 75 |

**Table S2**

*Characteristics of the Included Studies.*

| **Author,**  **Year, Country** | **Participant Characteristics** | | | **Intervention Characteristics** | | **Description of Control** | **Outcomes and Measures** | |
| --- | --- | --- | --- | --- | --- | --- | --- | --- |
|  | Type of patient | Sample Size (I/C) | Baseline Characteristics:  Age (years, mean, SD)  Sex (*n*, %) | Intervention Duration | Implementer |  | Measures and Outcomes | Data Collection Time-points |
| Cai, Zhao, et al., 2023 (China) | esophageal cancer | 91 (45/46) | -Age: 68.56 ± 9.55 (I),  66.17 ± 11.34 (C);  -Sex: male: 73 (80.22%),  female: 18 (19.78%); | 6 CALM sessions over 3 months (each session lasting 45~60 min and occurring every 2 weeks) | trained psychotherapists | usual treatment | ①psychological distress: DT  ②anxiety and depressive symptoms: HADS  ③Quality of Life: EORTC-QLQ-C30 | T0: Baseline or screening;  T1: The end of the intervention or control period (at 12 weeks);  T2: 1 month after the end of the intervention (at 16 weeks) |
| Wang et al., 2023 (China) | breast cancer | 103 (55/48) | -Age: 52.75 ± 7.799 (I),  51.15 ± 10 (C);  -Sex: not reported; | 6 CALM sessions over 4~6 months (Each session lasted 45 to 60 minutes, and the first three treatments were completed within the first month; subjects received a session once a month for 3 months) | psychologists and oncologist | usual care: included routine oncology treatment and follow-up | ①Fear of cancer recurrence: CWS  ②Quality of Life: FACT-B  ③Distress: DT  ④Anxiety and depression: HADS | T0: before CALM treatment;  T1: after 2 CALM sessions;  T2: after 4 CALM sessions;  T3: after 6 CALM sessions |
| Cai, Zhang, et al., 2023 (China) | gastrointestinal cancer | 126 (61/65) | -Age: 66.39 ± 10.73 (I),  64.38 ± 11.87 (C);  -Sex: male: 96 (76.19%),  Female: 30 (23.81%); | 6 CALM sessions over 3 months (each session lasting 60~70 min and occurring every 2 weeks) | trained psychotherapists | usual care: usual health education and stress relief for the same frequency and duration as participants in the CALM group | ①perceived fatigue: PFS-R  ②quality of life: EORTC-QLQ-C30  ③sleep quality: PSQI | T0: at baseline;  T1: at 12 weeks (after 6 sessions of intervention) |
| Pang et al., 2023 (China) | breast cancer | 60 (30/30) | -Age: 51.07 ± 8.63 (I),  52.93 ± 7.52 (C);  -Sex: not reported; | 6 CALM sessions over 3 months (each session lasting 45~60 min and occurring every 2 weeks) | psychologists and oncologist | usual care | ①sleep quality: SQS  ②psychological distress: DT  ③quality of life: The QOL scale | T0: at baseline;  T1: after the 12 weeks (after 6 sessions of intervention) |
| Zhao et al., 2023 (China) | lung cancer | 81 (40/41) | -Age: 62.05 ± 8.676 (I),  64.29 ± 9.328 (C);  -Sex: male: 55 (67.90%),  Female: 26 (32.10%); | 6 CALM sessions over 4 months (each session lasting 30 min, subjects received a session once a month for 3 months) | trained therapists including psychologist, oncologist, postgraduates | usual care | ①Fear of cancer recurrence: FCRI  ②quality of life: EORTC QLQ-C30 | T0: at baseline;  T1: immediately after treatment;  T2: 2 months after treatment;  T3: 4 months after treatment |
| Liu et al., 2023 (China) | early-stage breast cancer | 124 (62/62) | -Age: 49.26 ± 10.116 (I),  50.44 ± 8.586 (C);  -Sex: not reported; | 6 CALM sessions over 3 months (each session lasting 30 min) | trained therapists including psychologist, oncologist, postgraduates | standard care | ①quality of life: FACT-B  ②anxiety and depression levels: HADS | T0: at baseline;  T1: at the 6-week;  T2:at the12 weeks;  T3: at the 24 weeks |
| Jing et al., 2022 (China) | gastrointestinal cancer | 115 (61/54) | -Age: 66.8 ± 10.8 (I),  65.7 ± 9.0 (C);  -Sex: male: 83 (72.17%),  Female: 32 (27.83%); | 6 CALM sessions over 8 months (each session lasting 30 min, subjects received a session once a month for 3 months) | trained therapists including psychologist, oncologist, postgraduates | usual care | ①cancer-related fatigue: PFS  ②quality of life: EORTC QLQ-C30 | T0: at baseline  T1: after 2 sessions  T2: after 4 sessions  T3: after 6 sessions |
| Zhang et al., 2022 (China) | breast cancer | 77 (38/39) | -Age: 52.29 ± 7.686 (I),  51.03 ± 7.979 (C);  -Sex: not reported; | 6 CALM sessions over 3 months (each session lasting 30 min, and the first 3 treatments would be completed in the first month) | trained therapists including psychologist, oncologist, postgraduates | usual care | ①quality of life: FACT-B  ②psychological distress: DT  ③Fatigue: PFS  ④Somnipathy: PSQI  ⑤Anxiety: SAS  ⑥Depression: SDS  ⑦concern about recurrence: CARS | T0: at baseline  T1: after the Intervention |
| Mehnert et al., 2020 (Germany) | advanced cancer | 206 (99/107) | -Age: 59.50 ± 12.10 (I),  56.5 ± 11.3 (C);  -Sex: male: 80 (38.83%),  Female: 126 (61.17%); | 6 CALM sessions over 6 months and two optional booster sessions (each session lasting 50 min) | trained psychotherapists | non-manualized supportive psycho-oncological counselling intervention (SPI) | ①depression severity: BDI-II and PHQ-9  ②psychological distress: DT  ③Fatigue: BFI  ④Quality of life: QUAL-EC  ⑤Anxiety: GAD-7 | T0: at baseline  T1: 3 months after randomization  T2: 6 months after randomization |
| Ding et al., 2020 (China) | breast cancer | 74 (34/40) | -Age: 51.00 ± 6.950 (I),  50.65 ± 6.612 (C);  -Sex: not reported; | 3~6 CALM sessions over 3~6 months (each session lasting 30 min, subjects received 3 sessions within 3 months) | trained therapists including postgraduates, psychologist, oncologist | usual care | ①distress: DT  ②multidimensional QOL: FACT-B | T0: at baseline  T1: 1 month after treatment |
| Rodin et al., 2018 (Canada) | advanced cancer | 227 (107/120) | -Age: 59.05 ± 10.55 (I),  59.10 ± 11.48 (C);  -Sex: male: 107 (60.76%),  Female: 120 (39.24%); | 3~6 CALM sessions over 3~6 months (each session lasting 45~60 min) | master’s degree-level social workers and psychiatrists | usual care | ①concordant depression: PHQ-9  ②generalized anxiety symptoms: GAD-7 | T0: at baseline  T1: 3 months (primary end point)  T2: and 6 months (trial end point) |

**I/C:** intervention group/control group; **RCT:** randomized controlled trail; **CALM:** managing cancer and living meaningfully

**BFI:** Brief Fatigue Inventory; **CARS:** the Concerns About Recurrence Scale; **EORTC-QLQ-C30:** the European Organization for Research and Treatment of Cancer Quality of Life Questionnaire Core 30; **FACT-B:** the Functional Assessment of Cancer Therapy-Breast; **FCRI:** the Fear of Cancer Recurrence Inventory; **GAD-7:** Generalized Anxiety Disorder Questionnaire; **PFS:** the Piper Fatigue Scale; **PFS-R:** the Revised Piper Fatigue Scale; **PSQI:** the Sleep Quality Index scale; **QUAL-EC:** the Quality of Life at the End of Life Cancer Scale; **SAS:** the Self-Rating Anxiety Scale; **SDS:** the Self-Rating Depression Scale; **SQS:** the Sleep Quality Scale

**T0:** measurement at baseline; **T1:** First measurement; **T2:** Second measurement; **T3:** Third measurement

**Table S3**

*Supporting Evidence of the Cochrane Risk of Bias.*

| **No.** | **Study** | **Selection bias (random sequence generation)** | | **Selection bias (allocation concealment)** | | **Performance bias** | | **Detection bias** | | **Attrition bias** | | **Reporting bias** | | **Other bias** | |
| --- | --- | --- | --- | --- | --- | --- | --- | --- | --- | --- | --- | --- | --- | --- | --- |
|  |  | **Judge** | **Supports** | **Judge** | **Supports** | **Judge** | **Supports** | **Judge** | **Supports** | **Judge** | **Supports** | **Judge** | **Supports** | **Judge** | **Supports** |
| 1 | Cai, Zhao, et al., 2023 (China) | Unclear | No-details about random method | Unclear | No-details about allocation method | Unclear | No-details about blinding of participants and  personnel | Unclear | No-details about blinding of outcome assessors | Low | Multiple interpolation was used to analyze missing data at follow-up | Unclear | The study protocol is not available | Unclear | No-details about other significant bias risks |
| 2 | Wang et al., 2023 (China) | Unclear | No-details about random method | Unclear | No-details about allocation method | Unclear | No-details about blinding of participants and  personnel | Unclear | No-details about blinding of outcome assessors | Low | The number and reasons of missing data is comparable in each group | Unclear | The study protocol is not available | Unclear | No-details about other significant bias risks |
| 3 | Cai, Zhang, et al., 2023 (China) | Low | Computer-generated randomization | Low | Randomly group using sealed envelopes | High | Not concealing from patients, interventionist | Low | Blind to outcome assessors | Low | The number and reasons of missing data is comparable in each group | Unclear | The study protocol is not available | Unclear | No-details about other significant bias risks |
| 4 | Pang et al., 2023 (China) | Unclear | No-details about random method | Unclear | No-details about allocation method | Unclear | No-details about blinding of participants and  personnel | Unclear | No-details about blinding of outcome assessors | Low | No missing data | Unclear | The study protocol is not available | Unclear | No-details about other significant bias risks |
| 5 | Zhao et al., 2023 (China) | Low | Computer-generated randomization | Low | Randomly group using sealed cards | High | Not concealing from patients, interventionist | High | Not concealing from outcome assessors | Low | The number and reasons of missing data is comparable in each group | Unclear | The study protocol is not available | Unclear | No-details about other significant bias risks |
| 6 | Liu et al., 2023 (China) | Low | Computer-generated randomization | Low | Randomly group using sealed envelopes | High | Not concealing from interventionist | High | Not concealing from outcome assessors | Low | No missing data | Unclear | The study protocol is not available | Unclear | No-details about other significant bias risks |
| 7 | Jing et al., 2022 (China) | Low | Computer-generated randomization | Low | Randomly group using sealed envelopes | High | Not concealing from patients, interventionist | High | Not concealing from outcome assessors | Low | The number and reasons of missing data is comparable in each group | Unclear | The study protocol is not available | Unclear | No-details about other significant bias risks |
| 9 | Zhang et al., 2022 (China) | Low | Computer-generated randomization | Low | Randomly group using sealed envelopes | High | Not concealing from patients, interventionist | High | Not concealing from outcome assessors | Unclear | Reasons for missing data not described | Unclear | The study protocol is not available | Unclear | No-details about other significant bias risks |
| 11 | Mehnert et al., 2020 (Germany) | Low | Computer-generated randomization | Low | Adopting centralized random allocation | High | Not concealing from interventionist | High | Not concealing from outcome assessors | Low | Reported missing data and conducted the intention-to-treat (ITT) analysis | Unclear | The study protocol is not available | Unclear | No-details about other significant bias risks |
| 12 | Ding et al., 2020 (China) | Low | Computer-generated randomization | Low | Randomly group using sealed envelopes | High | Not concealing from patients, interventionist | High | Not concealing from outcome assessors | Low | Reported missing data and conducted the intention-to-treat (ITT) analysis | Unclear | The study protocol is not available | Unclear | No-details about other significant bias risks |
| 14 | Rodin et al., 2018 (Canada) | Low | Computer-generated randomization | Low | Randomly group using sealed envelopes | High | Not concealing from patients, interventionist | High | Not concealing from outcome assessors | Low | Reported missing data and conducted the intention-to-treat (ITT) analysis | Low | Study protocol available and all pre-specified outcomes reported in pre-specified way | Low | No bias introduced from other sources |

**Table S4**

*Quality of Evidence, Identified By GRADE Approach.*

| **Outcomes** | **No. of studies** | **Certainty assessment** | | | | | | **Effect** | | **Certainty** |
| --- | --- | --- | --- | --- | --- | --- | --- | --- | --- | --- |
|  |  | **Study design** | **Risk of bias** | **Inconsistency** | **Indirectness** | **Imprecision** | **Other considerations** | **No. of individuals** | **Rate (95% CI)** |  |
| Distress | 6 | RCT | Serious^a^ | Serious^c^ | Not serious^e^ | Not serious^f^ | None | 522 | MD -2.43  95%CI (-3.99 to -0.86) | ⨁⨁◯◯ Low |
| Anxiety | 6 | RCT | Serious^a^ | Serious^c^ | Not serious^e^ | Not serious^f^ | None | 745 | SMD -1.06  95%CI (-1.78 to -0.34) | ⨁⨁◯◯ Low |
| Depression | 7 | RCT | Serious^a^ | Serious^c^ | Not serious^e^ | Not serious^f^ | None | 870 | SMD -0.65  95%CI (-1.13 to -0.17) | ⨁⨁◯◯ Low |
| Quality of life | 10 | RCT | Serious^a^ | Serious^c^ | Not serious^e^ | Not serious^f^ | None | 972 | SMD 1.44  95%CI (0.47 to 2.40) | ⨁⨁◯◯ Low |
| Fear of cancer recurrence | 3 | RCT | Serious^a^ | Serious^c^ | Not serious^e^ | Not serious^f^ | None | 261 | SMD -2.06  95%CI (-4.59 to 0.47) | ⨁⨁◯◯ Low |
| Cancer related fatigue | 4 | RCT | Serious^a^ | Serious^c^ | Not serious^e^ | Not serious^f^ | None | 441 | SMD -3.54  95%CI (-5.84 to -1.23) | ⨁⨁◯◯ Low |
| Sleep Quality | 3 | RCT | Serious^a^ | Serious^c^ | Not serious^e^ | Not serious^f^ | None | 263 | SMD -1.00  95%CI (-1.86 to -0.14) | ⨁⨁◯◯ Low |

CI: confidence interval; MD: mean difference; SMD: standardized mean difference

^a^ some of the studies were assessed as high risk of bias; ^b^ most of the studies were assessed as low risk of bias/ some concerns; ^c^ I^2^>50%; ^d^ I^2^ = 0%; ^e^ direct participants, interventions, and outcomes in experimental and control groups; ^f^ total sample size > 300; ^g^ total sample size < 200

**
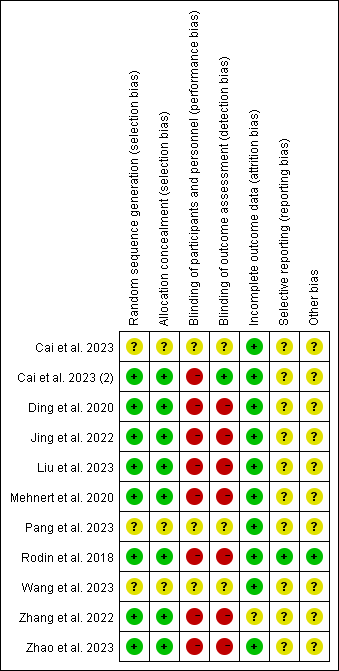

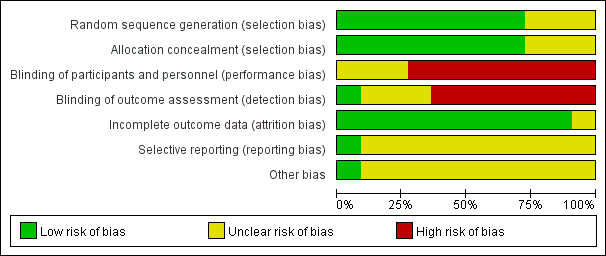
**

**Figure S1**

*The Results of Risk of Bias Assessment of included studies.*

| **(a)psychological distress**  **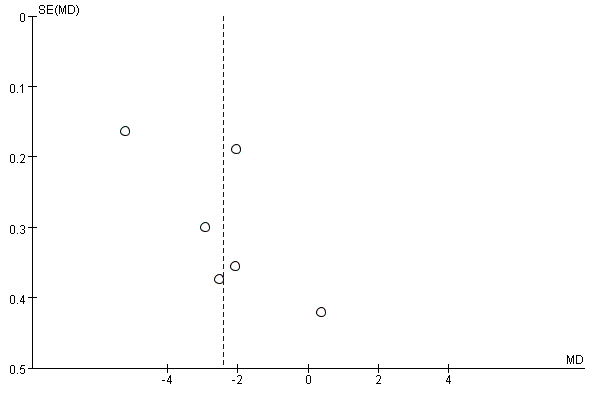** | **(b)Anxiety**  **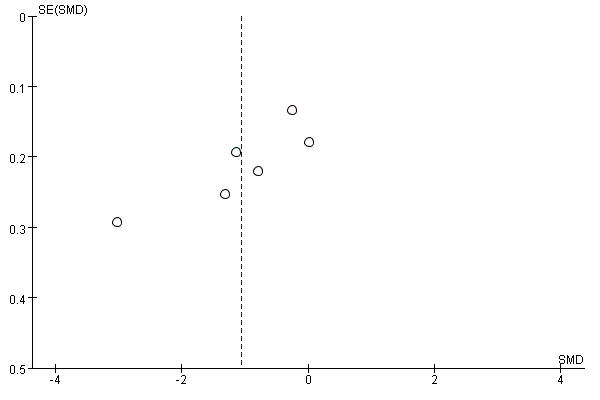** |
| --- | --- |
| **(c)Depression**  **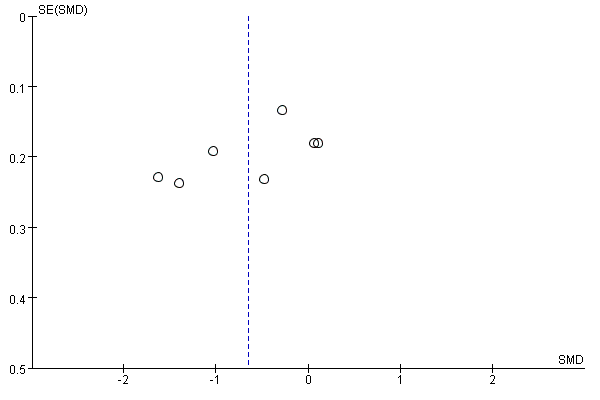** | **(d)Quality of life**  **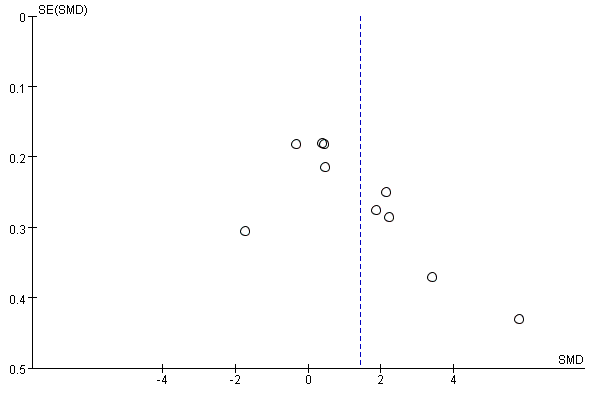** |

**Figure S2**

*Funnel plot for assessing publication bias.*
